# Vitamin D receptor ChIP-seq in primary CD4+ cells: relationship to serum 25-hydroxyvitamin D levels and autoimmune disease

**DOI:** 10.1186/1741-7015-11-163

**Published:** 2013-07-12

**Authors:** Adam E Handel, Geir K Sandve, Giulio Disanto, Antonio J Berlanga-Taylor, Giuseppe Gallone, Heather Hanwell, Finn Drabløs, Gavin Giovannoni, George C Ebers, Sreeram V Ramagopalan

**Affiliations:** 1 Medical Research Council Functional Genomics Unit and Department of Physiology, Anatomy and Genetics, University of Oxford, Parks Road, Oxford OX1 3PT, UK; 2 Blizard Institute, Queen Mary University of London, Barts and The London School of Medicine and Dentistry, 4 Newark Street, London E1 2AT, UK; 3 Department of Informatics, University of Oslo, Gaustadalléen 23 B, N-0373, Oslo, Norway; 4 Bioinformatics & Gene Regulation, Norwegian University of Science and Technology, Erling Skjalgsons gt. 1, N-7006, Trondheim, Norway

**Keywords:** Vitamin D, Autoimmune disease, ChIP-seq, Functional genomics

## Abstract

**Background:**

Vitamin D insufficiency has been implicated in autoimmunity. ChIP-seq experiments using immune cell lines have shown that vitamin D receptor (VDR) binding sites are enriched near regions of the genome associated with autoimmune diseases. We aimed to investigate VDR binding in primary CD4+ cells from healthy volunteers.

**Methods:**

We extracted CD4+ cells from nine healthy volunteers. Each sample underwent VDR ChIP-seq. Our results were analyzed in relation to published ChIP-seq and RNA-seq data in the Genomic HyperBrowser. We used MEMEChIP for *de novo* motif discovery. 25-Hydroxyvitamin D levels were measured using liquid chromatography–tandem mass spectrometry and samples were divided into vitamin D sufficient (25(OH)D ≥75 nmol/L) and insufficient/deficient (25(OH)D <75 nmol/L) groups.

**Results:**

We found that the amount of VDR binding is correlated with the serum level of 25-hydroxyvitamin D (r = 0.92, *P*= 0.0005). *In vivo* VDR binding sites are enriched for autoimmune disease associated loci, especially when 25-hydroxyvitamin D levels (25(OH)D) were sufficient (25(OH)D ≥75: 3.13-fold, *P*<0.0001; 25(OH)D <75: 2.76-fold, *P*<0.0001; 25(OH)D ≥75 enrichment versus 25(OH)D <75 enrichment: *P*= 0.0002). VDR binding was also enriched near genes associated specifically with T-regulatory and T-helper cells in the 25(OH)D ≥75 group. MEME ChIP did not identify any VDR-like motifs underlying our VDR ChIP-seq peaks.

**Conclusion:**

Our results show a direct correlation between *in vivo* 25-hydroxyvitamin D levels and the number of VDR binding sites, although our sample size is relatively small. Our study further implicates VDR binding as important in gene-environment interactions underlying the development of autoimmunity and provides a biological rationale for 25-hydroxyvitamin D sufficiency being based at 75 nmol/L. Our results also suggest that VDR binding in response to physiological levels of vitamin D occurs predominantly in a VDR motif-independent manner.

## Background

Vitamin D is a secosteroid produced from 7-dehydrocholesterol by the action of ultraviolet (UV) radiation within the skin and is hydroxylated to its active molecule 1,25-dihydroxyvitamin D (1,25D_3_) by the liver and kidneys [1]. A role for vitamin D and UV radiation in autoimmune disease was originally suggested by the latitudinal gradient in the prevalence and incidence for many autoimmune disorders [2]. Epidemiological studies have since confirmed the association of low levels of vitamin D with increased susceptibility to autoimmune disease, in some cases when vitamin D levels are measured prior to the clinical onset of disease [3-6]. The ideal dose of vitamin D supplementation to achieve a sufficient level of 25-hydroxyvitamin D is not clear, although it appears to be in excess of 800 international units [7].

1,25D_3_ acts intracellularly via the vitamin D receptor (VDR), a nuclear receptor that forms dimers with retinoid X receptors (RXR) to bind DNA and alter gene transcription [8]. Two studies have analyzed genome-wide binding of VDR using chromatin immunoprecipitation with massively parallel sequencing (ChIP-seq); one using a B-lymphoblastic cell line (LCL) and another using a monocytic cell line (MCL) [9,10]. The methods of vitamin D stimulation used in each study differed markedly and this may contribute to the differences in VDR binding observed in addition to cell-specific differences [11]. Each study determined that the VDR-RXR dimer recognizes a classical motif (DR3) but that this is present only at some of the VDR binding sites detected by ChIP-seq. The LCL ChIP-seq used genetic susceptibility *loci* drawn from genome-wide association studies to demonstrate significant overlap between autoimmune susceptibility regions and VDR binding sites [9].

However, *in vivo*, the situation is likely to be very different, both because DNA accessibility is likely to be altered in cell lines compared with primary immune cells and also because long-term exposure to physiological levels of 1,25D_3_ is not replicated well by short-term stimulation with high levels of 1,25D_3_[12-14]. In the present study we, therefore, aimed to use ChIP-seq to study VDR binding in primary CD4+ cells drawn from healthy individuals with measured serum levels of 25-hydroxyvitamin D.

## Methods

### Subjects

Healthy volunteers were recruited from the general public and nine samples of whole blood obtained (1_VDR, 2_VDR, 3_VDR, 4_VDR, 5_VDR, HB, PD, SP and SR). CD4+ lymphocytes were separated from whole blood using magnetic-activated cell sorting (MACS) as described in [15]. This project was approved by the Mid and South Buckinghamshire Research Ethics Committee (REC Reference # 09/H0607/7).

### 25-hydroxyvitamin D measurements

25-Hydroxyvitamin D was measured using liquid chromatography–tandem mass spectrometry.

### ChIP-seq

This was performed as in [9]. Briefly, CD4+ cells were fixed with 1% formaldehyde for 15 minutes then quenched with 0.125 M glycerine. Lysis buffer was added to isolate chromatin and the samples were disrupted with a Douce homogenizer. Sonication was used to sheer the resultant protein-DNA complex into 300 to 500 base pair fragments (Misonix, Farmindale, NY 11735, USA). DNA was quantified using a Nanodrop (Wilmington, DE 19810, USA) spectrophotometer.

Aliquots containing 50 μg of chromatin were precleared with protein A agarose beads (Invitrogen, Paisley PA4 9RF, UK). Genomic regions bound by VDR were precipitated out using anti-VDR rabbit antibody (Santa Cruz Biotechnology, sc-1008, Dallas, Texas 75220, USA) and isolated with protein A agarose beads. This was incubated at 4°C overnight, then washed and antibody-bound fragments eluted from the beads with SDS buffer. Samples were treated with proteinase K and RNase. Crosslinks were reversed by incubation overnight at 65°C. ChIP-DNA was purified by subsequent phenol-chloroform extraction and ethanol precipitation.

The purified product was then prepared for sequencing as per the Illumina ChIP-seq library generation protocol. The resultant DNA libraries were sent to Vanderbilt Microarray Shared Resource where they were sequenced on a Genome Analyzer II. Sequence reads (35 bases; 20 to 30 million quality filtered reads/sample) were aligned to the human genome (National Center for Biotechnology Information Build 37) using bowtie (0.10.1, [16], options ‘-n 2 -a —best —strata -m 1 -p 4’).

### ChIP-seq peak calling and artefact filtering

VDR ChIP-seq peaks were called using Zinba (zero-inflated negative binomial algorithm,refine peaks, extension = 200) with the false discovery rate set as <0.1% [17]. We removed peaks that showed overlap with regions known to give false positive ChIP-seq peaks by merging Terry’s blacklist and the list of ultra-high signal artefact regions [18]. ChIP-seq peaks are detailed in the (Additional file 1) dataset. We also called peaks separately using model-based analysis of ChIP-Seq (MACS) for further motif analysis [19].

### Motif analysis

MEME-ChIP [20], Weeder [21] and ChIPmunk [22] were used to identify *de novo* motifs from VDR ChIP-seq peaks from groups of samples with 25-hydroxyvitamin D <75 nM and ≥75 nM, intervals overlapping with LCL/MCL VDR ChIP-seq peaks and intervals overlapping with RXR ChIP-seq peaks from NB4 cells [20,23]. ChIP-seq peaks were also scanned for known VDR recognition motifs using RSAT [24] and Fimo [25].

### GREAT gene ontology analysis

25(OH)D ≥75 and 25(OH)D <75 VDR binding sites were input into the Genomic Regions Enrichment of Annotations Tool (GREAT) using the GRCh37 (UCSC hg19, February 2009) assembly and 5 kb proximal and 1 kb distal gene windows [26].

### Overlap and hierarchal clustering analysis

The Genomic HyperBrowser was used to determine overlap and hierarchal clustering between different datasets [27,28]. Autoimmune disease-associated regions were determined as those 100 kb either side of a SNP associated with an autoimmune disease in the Genome Wide Association Study Catalogue with a *P*-value ≤1×10^-7^[29] (downloaded 13 June 2012). Samples were combined into 25(OH)D ≥75 and 25(OH)D <75 by merging all binding sites from samples with 25-hydroxyvitamin D ≥75 nM (n = 5) and <75 nM (n = 4). Overlap was determined using segment-segment analysis with either 1,000 or 10,000 Monte-Carlo randomizations maintaining the empiric distribution of segment and inter-segment lengths, but randomizing positions. Controlling for gene or immune gene position (obtained from the Gene Ontology project [30]) used an intensity track created based on the proximity of (pooled) VDR regions to their nearest genes or immune genes, respectively. VDR regions were represented as points (midpoints of VDR binding peaks) and a point-segment analysis using 1,000 Monte-Carlo randomizations with points sampled according to the intensity track, were used to compute *P*-values (auto-immune regions represented as segments as before). Immune gene-controlled overlap omitted chromosome Y as no immune genes were located there. Comparisons between 25(OH)D <75 and 25(OH)D ≥75 for overlap were performed using case-control tracks generated by the Genomic HyperBrowser and analyzed using valued segment–segment preferential overlap analysis with 10,000 Monte-Carlo randomizations, keeping the location of segments of both tracks constant, while randomly permuting case-control values of the first track in the null model. Heirarchical clustering analysis was performed in the Genomic HyperBroswer by obtaining pairwise overlap-enrichment values for each of the samples and computing distance between samples as the inverse of these values. Th1 DNase I hypersensitivity peaks were obtained from the University of California at Santa Cruz (UCSC) Table Browser and were generated by the Duke group [31]. ChIP-seq peaks for VDR in LCLs and MCLs were obtained from previously published studies using VDR binding intervals after stimulation with calcitriol [9,10], and co-factor ChIP-seq peaks were obtained from the Encyclopedia of DNA Elements (ENCODE) and Cistrome, using ChIP-seq data from hematopoietic cell lines (GM121878, K562 and NB4) [23,31-33]. ChIP-seq data on chromatin states (H3K27Ac, H2A.Z, H3K4me1, H3K4me2, H3K4me3, H3K9Ac and H3K9me3) in GM12878 cells and chromatin looping 5C data were obtained from ENCODE [34,35]. Gene expression data from CD4+ cells was obtained from data published by Birzele and colleagues [36]. Gene expression data from LCLs in response to 1,25D_3_ treatment was obtained from Ramagopalan and colleagues [9].

## Results

### VDR binding sites in CD4+ cells

VDR binding in samples from nine individuals ranged from 200 to 7,118 binding sites across the genome. There was a significant correlation between measured 25-hydroxyvitamin D levels and the number of VDR binding sites (r = 0.92, *P*= 0.0005, Table 1).

**Table 1:** Number of VDR binding sites

**Sample/group**	**25-OH D**	**Total**	**Downstream**	**Exons**	**Intergenic**	**Introns**	**Up and downstream**	**Upstream**	**UTR**
		**Number**	**%**						
High 25-hydroxyvitamin D	≥75	13,054	5.3	4.0	32.8	28.4	4.7	14.6	10.2
Low 25-hydroxyvitamin D	<75	1,526	3.7	4.8	36.5	18.6	4.3	18.8	13.3
1_VDR	80	3,073	5.2	3.9	37.9	24.0	4.7	13.8	10.5
2_VDR	107	7,118	5.6	4.5	28.2	26.3	5.1	17.0	13.2
3_VDR	76	5,290	5.4	3.5	36.8	29.1	4.1	12.9	8.3
4_VDR	85	3,059	5.1	4.0	37.3	22.3	4.7	15.6	11.0
5_VDR	75	4,051	4.6	4.8	33.0	21.5	5.3	18.8	12.1
HB	29	1,021	3.5	5.7	27.4	16.9	5.6	24.1	17.0
PD	32	200	1.0	3.0	61.7	21.4	0.5	7.0	5.5
SP	34	610	3.2	4.5	38.7	18.8	3.7	18.3	13.0
SR	22	573	3.5	4.9	39.7	17.7	4.0	18.5	11.7
r		0.92	0.82	−0.22	−0.40	0.79	0.44	−0.14	−0.05
P		0.0005	0.0068	0.5746	0.2822	0.0115	0.2401	0.7163	0.891

The number of VDR binding sites at FDR <0.1%. 25-hydroxyvitamin D (25(OH)D) levels are shown in nM. Correlations are shown between 25(OH)D and the number of binding sites. VDR, vitamin D receptor.

**Figure 1:**
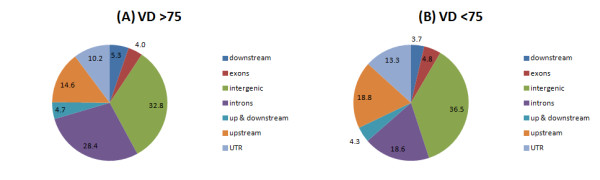
**Genomic regions of VDR binding sites.** The midpoints of each VDR ChIP-seq peak is shown for **(A)** samples with 25-hydroxyvitamin D ≥75 nM and **(B)** samples with 25-hydroxyvitamin D <75 nM. Up-, down- and up and downsteam are VDR binding sites within 5 kb of the nearest gene. Numbers show percentages of binding sites within each genomic region. ChIP-seq, chromatin immunoprecipitation and massively parallel sequencing; VDR, vitamin D receptor.

For the purposes of analysis we split our samples into two groups, one with sufficient 25-hydroxyvitamin D (25(OH)D ≥75 nM, n = 5, 3 men, 2 women, age range 20 to 30 years, mean 25(OH)D 84.6 nM, range 75 to 107) and one with 25-hydroxyvitamin D insufficiency/deficiency (25(OH)D <75 nM, n = 4, 2 men, 2 women, age range 24 to 32 years, mean 29.3 nM, range 22 to 34; 25-hydroxyvitamin D in 25(OH)D ≥75versus 25(OH)D <75*P*<0.05). Our cut-off of 75 nM is supported by recommended clinical guidelines [37]. The five samples with 25(OH)D ≥75 had many more VDR binding sites than the four samples with 25(OH)D <75 (25(OH)D ≥75 mean number of binding sites 4,518 (range 3,059 to 7,118); 25(OH)D <75 mean number of binding sites 601 (range 200 to 1,021); 25(OH)D ≥75 versus 25(OH)D <75 *P*= 0.02). The genomic regions at which VDR binding sites were found also differed with vitamin D level (Figure 1). This was predominantly driven by an increase in intronic VDR binding in 25(OH)D ≥75 samples. For individual samples, VDR binding within 5 kb downstream of genes (r = 0.82, *P*= 0.007) and within introns (r = 0.79, *P*= 0.01) was correlated with vitamin D levels, whereas VDR binding in areas with 5 kb upstream (r = -0.14, *P*= 0.72) or both upstream and downstream (r = 0.44, *P*= 0.24) of genes, within exons (r = -0.21, *P*= 0.57), UTRs (r = -0.05, *P*= 0.89) or intergenic regions (r = -0.40, *P*= 0.28) was not.

We performed hierarchical clustering analysis using pairwise overlap-enrichment of VDR binding sites and this revealed far closer similarity between samples within each group (25(OH)D ≥75 and 25(OH)D <75) than when comparing samples between groups [see Additional file 2: Figure S1]. Binding sites were also frequently shared between samples, but 66.0% of binding sites were unique to a single sample.

### VDR binding and gene ontology

VDR binding sites were assessed for overlap with known gene ontology biological pathways in GREAT [See Additional file 3: Table S1] [26]. In 25(OH)D ≥75 samples, binding sites were maximally enriched for pathways involved in RNA processing, gene expression, protein folding and T cell activation or differentiation. In contrast, the top pathways enriched for 25(OH)D <75 VDR binding were involved in RNA splicing, translation and histone modification.

### VDR binding motifs

We found that there was no significant enrichment of binding sites containing DR3-like motifs either when searching *de novo* using MEME-ChIP [20], CentriMo [38], Weeder [21] or ChIPmunk [22] and analyzing all binding sites, binding sites grouped by high or low vitamin D, binding sites overlapping with the previous LCL or MCL VDR ChIP-seq studies, binding sites common between multiple samples or binding sites overlapping with previous ChIP-seq studies of RXR in NB4 cells [23]. Neither were DR3-like motifs found when each sample was analyzed independently. The top consensus binding sites are shown in Additional file 4: Figure S2 for each analysis approach. Our methods were, however, able to detect the reported DR3 sites in previous VDR ChIP-seq studies [9,10]. We were also unable to detect VDR-like motifs when restricting our search to only those parts of ChIP-seq intervals common to all samples in the 25(OH)D ≥75 or 25(OH)D <75 groups.

As this was an unexpected finding, we performed an *in silico* search within the pooled peaks but did not identify an over-representation of known VDR binding motifs using RSAT [24] and Fimo [25]. The existing RXRA::VDR motif in the Jaspar [39] and TRANSFAC [40] databases has been generated from SELEX data, which mainly will represent strong binding without additional co-factors or other context-dependent features. It is, therefore, relevant to search for alternative variants of VDR-like motifs that may be more representative of *in vivo* binding. Since the CD4+ data set, in particular, shows a lack of centrally enriched binding site motifs, MEME-ChIP and CentriMo are less suitable for this. Therefore, an iterative approach was used, in which the full set of ChIP-Seq regions for LCL, MCL and the merged set of CD4+ regions was searched with MAST and the RXRA::VDR matrix (*P*-value 0.0001, E-value 100.0) [41]. The significant regions were submitted to MEME for *de novo* motif discovery. In each data set a VDR-like motif was found. This motif was used as input to MAST again, and the resulting positive set was submitted to MEME, in order to reduce bias from the original RXRA::VDR motif. This process can, in principle, be repeated several times, but in most cases the motifs will start to degenerate after a while into very general motifs with low information content. However, the motifs generated in this case are clearly similar to the classical RXRA::VDR motif, although with distinct differences [See Additional file 5: Figure S3]. They are also similar to the previously published motifs for LCL and MCL. These improved matrices were then used with MAST to make positive and negative subsets for further analysis. Here a slightly higher *P*-value was used (0.0005) in order to include more borderline motifs, leading to 811 positive sequences (29%) for LCL, 648 (28%) for MCL, and 90 (0.4%) for CD4+. This seems to confirm the lack of VDR-like motifs in the CD4+ set. This was further confirmed using FIMO to search each data set with both the RXRA::VDR matrix and the individually optimized matrices generated above [See Additional file 6: Figure S4]. This showed a clear lack of significant motifs in the CD4+ data, independent of which matrix was used for searching. Analyzing CD4+ binding intervals for other JASPAR motifs showed only a significant overrepresentation of CTCF binding motifs in the 25(OH)D ≥75 but not 25(OH)D <75 group.

We found significant overlap between CD4+ VDR and RXR ChIP-seq peaks drawn from a promyelocytic cell line (NB4; Additional file 7: Table S2) (25(OH)D ≥75 19.77-fold, *P*= 0.0004; 25(OH)D <75 65.14-fold, *P*<0.0001 [23]) and significant overlap between VDR binding sites in CD4+ cells and those observed previously in LCLs (25(OH)D ≥75 70-fold, *P*<0.0001; 25(OH)D <75 151.7-fold, *P*<0.0001; 813/2,776 (29.3%) LCL VDR binding sites overlap with VDR binding sites in CD4+ cells) and MCLs (25(OH)D ≥75 28.75-fold, *P*<0.0001; 25(OH)D <75 37.17-fold, *P*<0.0001; 353/1,818 (19.4%) MCL VDR binding sites overlap with VDR binding sites in CD4+ cells) making it likely that our data reflect real VDR binding sites.

Motifless binding has been described by the ENCODE project with characteristically greater enrichment of DNase I hypersensitivity than binding sites with classical motifs [35]. We confirmed this in the previous LCL and MCL VDR ChIP-seq datasets by dividing binding sites into those with or without a VDR-like motif as detailed above. Intervals containing the VDR-like motif had less enrichment of DNase I peaks in GM12878 LCLs than those intervals lacking that motif (LCL peaks with a VDR-like motif (LCL_motif_), 24.6-fold, *P*<0.0001; LCL peaks without a VDR-like motif (LCL_no motif_), 27.8-fold, *P*<0.0001; LCL_motif_ versus LCL_no motif_*P*= 0.0002; MCL_motif,_ 13.5-fold, *P*<0.0001; MCL_no motif_, 18.0-fold, *P*<0.0001; MCL_motif_ versus MCL_no motif_*P*= 0.0002). VDR ChIP-seq peaks in the CD4+ cells in this study overlapped more with binding sites in LCLs and MCLs lacking binding motifs than those with motifs (LCL_motif_ 37.4-fold, *P*<0.0001; LCL_no motif_ 79.4-fold, *P*<0.0001; LCL_motif_ versus LCL_no motif_*P*= 0.0002; MCL_motif,_ 17.7-fold, *P*<0.0001; MCL_no motif_, 32.3-fold, *P*<0.0001; MCL_motif_ versus MCL_no motif_*P*= 0.0002).

### VDR co-factors, chromatin state and calcitriol-responsive gene expression

**Figure 2:**
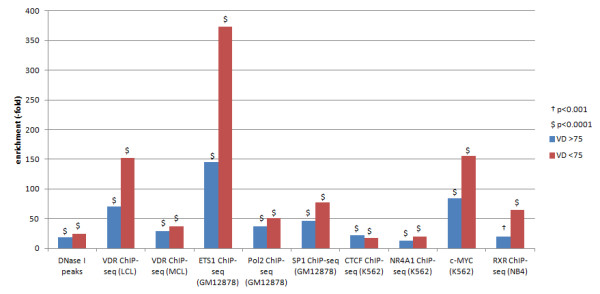
**Overlap of VDR ChIP-seq peaks with genomic features.** 25(OH)D ≥75, samples with 25-hydroxyvitamin D ≥75 nM; 25(OH)D <75, samples with 25-hydroxyvitamin D <75 nM; ChIP-seq, chromatin immunoprecipitation and massively parallel sequencing; LCL, lymphoblastoid cell line; MCL, monocytic cell line; VDR, vitamin D receptor.

We found significant overlap between the known VDR co-factors SP1 in GM12878 cells (VD ≥75 45.86-fold, *P*<0.0001; 25(OH)D <75 76.8-fold, *P*<0.0001), ETS1 in GM12878 cells (25(OH)D ≥75 145.4-fold, *P*<0.0001; 25(OH)D <75 373.5-fold, *P*<0.0001), NR4A1 in K562 cells (25(OH)D ≥75 12.5-fold, *P*<0.0001; 25(OH)D <75 19.4-fold, *P*<0.0001) and c-MYC in K562 cells (25(OH)D ≥75 83.9-fold, *P*<0.0001; 25(OH)D <75 155.4-fold, *P*<0.0001). ChIP-seq data were from the UCSC Genome Browser and our VDR binding sites [See Additional file 7: Table S2; Figure 2] [31]. Given our finding that some VDR ChIP-seq peaks were enriched for CTCF motifs, we analyzed overlap with known CTCF binding intervals in K562 cells and again found significant overlap (25(OH)D ≥75 22.26-fold, *P*<0.0001; 25(OH)D <75 17.16-fold, *P*<0.0001). There was also significant overlap with open chromatin in T_h1_ cells, as determined by DNase I hypersensitivity regions (25(OH)D ≥75 18.93-fold, *P*<0.0001; 25(OH)D <75 23.71-fold, *P*<0.0001). For each of these analyses apart from CTCF, 25(OH)D <75 was significantly more enriched for the tested genomic features than 25(OH)D ≥ 5 [See Additional file 7: Table S2].

**Figure 3:**
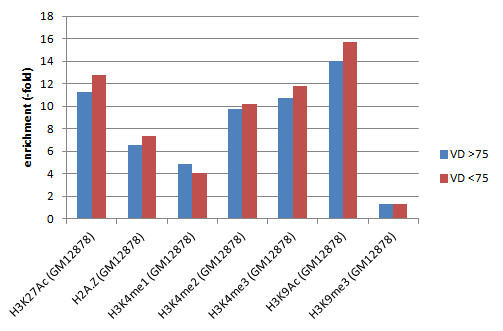
**Overlap of VDR ChIP-seq peaks with chromatin marks.** 25(OH)D ≥75, samples with 25-hydroxyvitamin D ≥75 nM; 25(OH)D <75, samples with 25-hydroxyvitamin D <75 nM; ChIP-seq, chromatin immunoprecipitation and massively parallel sequencing; VDR, vitamin D receptor. All bars shown are significant at *P*<0.0001.

VDR ChIP-seq peaks showed the highest enrichment for chromatin marks in GM12878 cells associated with transcriptional regulation (H3K27Ac, H2A.Z, H3K4me1, H3K4me2, H3K4me3 and H3K9Ac) and far lower enrichment for a repressive chromatin mark (H3K9me3) [See Additional file 7: Table S2; Figure 3] [35].

There was significant enrichment of VDR binding within 5 kb of genes responsive to 1,25D_3_ treatment detected from microarray expression data in LCLs (25(OH)D ≥75 3.86-fold, *P*<0.0001; 25(OH)D <75 2.98-fold, *P*= 0.0002; 25(OH)D ≥75 versus 25(OH)D <75 *P*= 0.004) [9].

Given the relatively high proportion of intergenic VDR binding sites, we tested for overlap with sites of known chromatin looping in GM12878 cells in pilot ENCODE regions [34]. There was significant but low magnitude overlap of VDR binding and chromatin looping in 25(OH)D ≥75 samples but not 25(OH)D <75 samples (25(OH)D ≥75 1.07-fold, *P*= 0.002; 25(OH)D <75 0.73-fold, *P*= 0.83; 25(OH)D ≥75 versus 25(OH)D <75 *P*= 0.01).

### VDR binding sites and autoimmune disease

**Figure 4:**
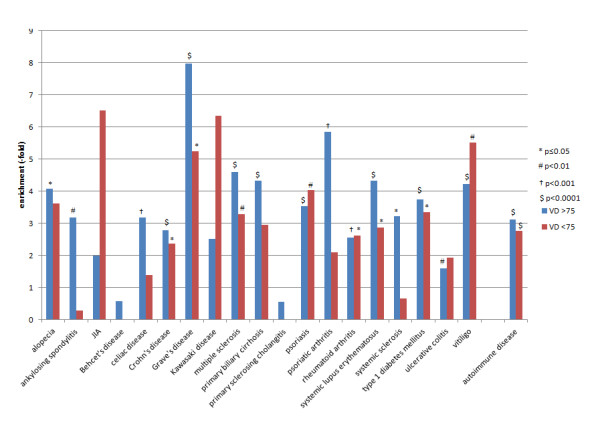
**Overlap of VDR ChIP-seq peaks with autoimmune disease-associated regions.** Autoimmune disease associated regions are those within 100 kb of SNPs implicated in genome-wide association studies (GWAS) at *P*<10^-7^[29]. 25(OH)D ≥75,samples with 25-hydroxyvitamin D ≥75 nM, 25(OH)D <75, samples with 25-hydroxyvitamin D <75 nM; ChIP, ChIP-seq, chromatin immunoprecipitation and massively parallel sequencing; VDR, vitamin D receptor.

We assessed overlap between VDR ChIP-seq peaks and genomic regions encompassing the area 100 kb around SNPs significantly associated with autoimmune disease in genome wide association studies [29]. There was a significant enrichment within all regions associated with autoimmunity and this was greater for 25(OH)D ≥75 than 25(OH)D <75 (25(OH)D ≥75: 3.13-fold, *P*<0.0001; 25(OH)D <75: 2.76-fold, *P*<0.0001; 25(OH)D ≥75 enrichment versus 25(OH)D <75 enrichment: *P*= 0.0002). Overlap for individual autoimmune diseases is detailed in Additional file 8: Table S3 and illustrated in Figure 4. There was significant overlap for alopecia, ankylosing spondylitis, celiac disease, Crohn’s disease, Grave’s disease, multiple sclerosis, primary biliary cirrhosis, psoriasis, psoriatic arthritis, rheumatoid arthritis, systemic lupus erythematosus, systemic sclerosis, type 1 diabetes mellitus, ulcerative colitis and vitiligo. In most conditions, there was more overlap for 25(OH)D ≥75 than 25(OH)D <75. One possible explanation would be that both VDR binding and autoimmune disease regions tend to cluster near regions enriched for genes so the analysis was repeated controlling for the location of genes and immune-related genes. Controlling for immune-related genes reduced the significance for some autoimmune diseases (notably rheumatoid arthritis) suggesting that VDR binding near immune-genes may underlie some of the enrichment seen near autoimmune disease regions. However, overall overlap with autoimmune disease regions was still significant suggesting that VDR enrichment of these regions is at least partially independent of preferential binding near immune-related genes. We assessed enrichment for autoimmune disease-associated regions in all VDR binding sites overlapping with ChIP-seq peaks for other transcription factors and found the greatest enrichment for overlap with SP1 and CTCF but comparisons between VDR binding sites overlapping with transcription factor ChIP-seq peaks and those without overlap were not significant [See Additional file 9: Table S5].

There was no significant enrichment for genomic regions associated with control conditions (ones in which CD4+ cells would not be expected to play a dominant role), such as coronary heart disease, atopic dermatitis and type 2 diabetes mellitus (*P*>0.05 for all). Also, in support of separate biochemical pathways for autoimmunity and metabolic effects of vitamin D, VDR binding was not enriched for genomic regions associated with bone mineral density.

The previous study of LCLs had shown VDR enrichment near regions associated with chronic lymphocytic leukemia. However, no significant enrichment was seen for these regions in primary CD4+ cells (25(OH)D ≥75 1.62-fold, *P*= 0.37; 25(OH)D <75 2.44-fold, *P*= 0.27; LCLs 20.7-fold, *P*<0.0001), suggesting that VDR binding in cell lines differs considerably from that seen in primary immune cells.

Although 100 kb was chosen to encompass the likely extent of linkage disequilibrium,both groups showed increased enrichment when the size of the region assessed for overlap decreased. 25(OH)D ≥75 showed consistently greater enrichment for autoimmune regions than 25(OH)D <75 [See Additional file 10: Figure S5].

Several disease-associated SNPs were located within VDR ChIP-seq binding intervals [See Additional file 11: Table S4]. We analyzed these SNPs in Regulome DB and found that several were likely to affect gene expression and/or transcription factor binding [42].

### VDR binding and gene expression in CD4+ cells

We assessed enrichment in VDR binding near genes expressed in different types of CD4+ cells measured by RNA-seq [36]. VDR binding was significantly enriched within 5 kb of genes expressed either specifically in T-regulatory cells or T-helper cells and genes expressed which were common to all CD4+ cells. Enrichment was particularly high for genes associated specifically with T-regulatory and T-helper cells in the 25(OH)D ≥75 group (RNA-seq T_reg_: 25(OH)D ≥75 4.07-fold, *P*<0.0001; 25(OH)D <75 2.96-fold, *P*<0.0001; 25(OH)D ≥75 versus 25(OH)D <75 *P*= 0.0002; RNA-seq T_helper_: 25(OH)D ≥75 3.87-fold, *P*<0.0001; 25(OH)D <75 2.76-fold, *P*<0.0001; 25(OH)D ≥75 versus 25(OH)D <75 *P*= 0.0002; RNA-seq CD4 + _common_: 25(OH)D ≥75 5.27-fold, *P*<0.0001; 25(OH)D <75 5.13-fold, *P*<0.0001; 25(OH)D ≥75 versus 25(OH)D <75 *P*= 0.0002).

## Discussion

The most arresting finding in this study is that the number of VDR binding sites in primary CD4+ cells is strongly correlated with 25-hydroxyvitamin D levels. The previous VDR ChIP-seq experiments using MCLs and LCLs found an increase in VDR binding site occupancy following treatment with supraphysiological levels of calcitriol [9,10]. Our finding of a far greater number of VDR binding sites in sufficient vitamin D samples than insufficient samples suggests that this effect also occurs with different *in vivo* levels of vitamin D. *In vivo* levels of 25-hydroxyvitamin D are directly associated with the number of VDR binding sites.

VDR binding sites are enriched for markers of active transcription and open chromatin; 25(OH)D ≥75 samples seemed to be less enriched for these markers than 25(OH)D <75, perhaps reflecting binding to open chromatin state in 25(OH)D <75 samples.

We have confirmed that the observation of significant overlap between VDR binding and genomic regions implicated in autoimmune diseases in LCLs is also seen in primary CD4+ cells [9,10]. Gene ontology analysis suggests that VDR binding in conditions of 25-hydroxyvitamin D sufficiency may be more directly related to immune cell function. This is supported by the observed higher levels of VDR binding near genes expressed specifically in T-regulatory and T-helper cells in 25(OH)D ≥75 but not 25(OH)D <75 samples.

We found a lack of classical VDR binding motifs within the VDR ChIP-seq peaks. In the ChIP-seq studies in MCLs and LCLs the authors identified classical DR3 motifs at differing proportions of sites (32% in MCLs, 67% in LCLs) with SP1-like and ETS-like non-classical peaks identified in the MCL ChIP-seq study (23% and 12% respectively) [9,10]. We found enrichment of CTCF motifs in several of our samples but were unable to identify any previously described VDR motifs. One possibility is that *in vivo* VDR binding is modulated by protein-protein interactions with co-factors: SP1 and ETS1 are known to modulate VDR binding, and there is some evidence that interactions between SP1 and VDR may enable modulation of genes that lack a classical VDR recognition motif [43,44]. Several other proteins are known to bind in association with VDR, including NR4A1 and c-MYC [45,46]. CTCF is known to modulate DNA binding via protein-protein interactions with other nuclear receptors [47-49]. However, it is unlikely that protein-protein interactions with transcription factors with specific recognition sequences can explain most of these motifless binding sites since one would have expected to find that motif through MEME-ChIP analysis. It may be that in response to physiological levels of 25-hydroxyvitamin D most VDR binding occurs at motifless binding sites similar to those identified by ENCODE [35], supported by the increased overlap with DNase I peaks. Another possibility is that the lack of motifs may reflect the fact that these CD4+ cells were not stimulated with 1,25D_3_, as the previous LCL ChIP-seq did not find classical motifs prior to stimulation [9]. Alternatively, current motif-finding methods may be insufficient to locate true VDR binding motifs. Further research will be needed in more lymphocyte subsets to delineate further the role of non-classical binding sites in VDR binding. It would also be useful to obtain 1,25D_3_, parathyroid hormone and calcium measurements for future study.

The overlap between genomic regions associated with many autoimmune diseases and VDR binding in primary CD4+ cells strongly suggests a role for vitamin D in many of these diseases, as already seen for MCLs and LCLs [9,10]. This is strengthened by the observation that this effect tends to be stronger in individuals sufficient for 25-hydroxyvitamin D. Interestingly, the magnitude of enrichment for autoimmunity increased as the flanks of the region surrounding implicated SNPs was reduced. This further suggests that this is not a chance finding and that VDR binding may have a functional role in modulating adaptive immunity in autoimmune diseases. We also controlled for genomic architectural features that could bias our results and observed that the results were not substantively altered. Future functional work should focus on the effects of VDR binding on nearby gene expression and targeted sequencing in patients with autoimmune conditions to identify possible rare variants affecting VDR binding.

## Conclusions

The role of vitamin D in bone health has long been established. The involvement of this vitamin in autoimmune disease is however heavily debated. We provide here an *in vivo* mechanism as to how vitamin D deficiency may influence autoimmune disease risk, by directly interacting with disease associated genes. Vitamin D sufficiency has been suggested to have a threshold of approximately 75 nmol/L; we provide here biological evidence in support of this, with significant public health implications.

## Consent

All subjects gave written informed consent for their samples to be used in this study.

## Abbreviations

1,25D3: 1,25-dihydroxyvitamin D; 25-OH D: 25-hydroxyvitamin D; ChIP-seq: Chromatin immunoprecipitation and massively parallel sequencing; ENCODE: Encyclopedia of DNA Elements; LCL: Lymphoblastoid cell line; MACS: Magnetic activated cell sorting; MCL: Monocytic cell line; RXR: Retinoid X receptors; SNP: Single nucleotide polymorphism; UTR: Untranslated region; VDR: Vitamin D receptor; 25(OH)D ≥ 75: Samples with 25-hydroxyvitamin D ≥75 nM; 25(OH)D <75: Samples with 25-hydroxyvitamin D <75 nM.

## Competing interests

The authors declare that they have no competing interests.

## Authors’ contributions

AEH, GD and SVR designed the study; AEH, GS and GD collected the data; AEH, GD, GS, FD and SVR analyzed the data; AEH, GS, GD, AJB-T, GG, HH, FD, GGiovannoni, GCE and SVR wrote the paper and critically revised its contents. All authors read and approved the final manuscript.

## Pre-publication history

The pre-publication history for this paper can be accessed here:

http://www.biomedcentral.com/1741-7015/11/163/prepub

## Supplementary Material

Additional file 1 **Supplementary dataset.** VDR binding sites in samples with high and low 25-hydroxyvitamin D. Genomic co-ordinates are shown in hg19. 25(OH)D≥75 = samples with 25-hydroxyvitamin D ≥75 nM, 25(OH)D<75 = samples with 25-hydroxyvitamin D <75 nM. Click here for file

Additional file 2: Figure S1 Heirarchical clustering of VDR ChIP-seq peaks for individual samples. **(A)** Distance matrix computing distances as the inverse of overlap-enrichment pairwise similarity between samples (color scheme ranges from red for most similar to white for least similar). **(B)** Dendrogram incorporating the distances from the distance matrix. V1-5, VDR_1 to VDR_5; 25-hydroxyvitamin D ≥75 nM. HB, PD, SP and SR, 25-hydroxyvitamin D <75 nM. Click here for file

Additional file 3: Table S1 Top gene ontology terms by binomial FDR Q-value. Values are derived from GREAT. 25(OH)D≥75 = subjects with vitamin D levels ≥75 nM, 25(OH)D<75 = subjects with vitamin D levels <75 nM. Click here for file

Additional file 4: Figure S2 Top MEME-ChIP motifs for VDR ChIP-seq peaks. This figure shows the top two motifs for each set of VDR ChIP-seq peaks by E-score as established by MEME-ChIP [15]: VD ≥ 75, samples with 25-hydroxyvitamin D ≥75 nM; VD <75, samples with 25-hydroxyvitamin D <75 nM. CD4+ VDR ChIP-seq peaks overlapping LCL VDR ChIP-seq peaks and CD4+ VDR ChIP-seq peaks overlapping NB4 RXR ChIP-seq peaks. Click here for file

Additional file 5: Figure S3 Sequence logos for VDR-like binding site motifs. The motifs were identified for each data set by searching the full ChIP-Seq regions with the Jaspar/TRANSFAC RXRA::VDR motif using MAST, followed by *de novo* motif discovery with MEME on the positive regions from MAST [15]. The resulting VDR-like matrix was used for another round of MAST searching on the full ChIP-Seq regions and MEME motif discovery on the positive set. The final matrices are shown for **(A)** LCL (434 sites used by MEME), **(B)** MCL (288 sites), **(C)** CD4+ (56 sites), and **(D)** Jaspar/TRANSFAC RXRA::VDR. The observation that the LCL and MCL logos are more similar to each other than to the RXRA::VDR logo, whereas the logo for CD4+ is more similar to the RXRA::VDR logo, may reflect the fact that the two first logos are based on a much larger number of sites and are, therefore, more likely to represent the true binding site motif for strong binding. Click here for file

Additional file 6: Figure S4 Number of sequences retrieved from each data set by motif-based searches. Motif occurrences were identified using FIMO with **(A)** the RXRA::VDR motif, or **(B)** individually optimal matrices for each data set (LCL, MCL and CD4+) [20]. The number of sequences with at least one motif is plotted as a function of motif *P*-value. Each *P*-value is corrected for data set size by multiplying it with the number of tests. Click here for file

Additional file 7: Table S2 Enrichment of genomic features within VDR binding sites. This shows the enrichment within VDR binding intervals for VDR binding intervals in lymphoblastoid cell lines (LCL VDR) and monocytic cell lines (MCL VDR), and other genomic features drawn from Cistrome and ENCODE [18,25,44]. 25(OH)D≥75 = subjects with vitamin D levels ≥75 nM, 25(OH)D<75 = subjects with vitamin D levels <75 nM, O/E = observed/expected overlap of genomic intervals, p = p-value calculated from 10,000 Monte-Carlo randomisations. Click here for file

Additional file 8: Table S3 Enrichment of autoimmune disease susceptibility regions within VDR binding sites. Autoimmune susceptibility regions were defined as those within 100kb of SNPs associated with autoimmune disease [27]. 25(OH)D≥75 = subjects with vitamin D levels ≥75 nM, 25(OH)D<75 = subjects with vitamin D levels <75 nM, O/E = observed/expected overlap of genomic intervals, p = p-value calculated from 10,000 Monte-Carlo randomisations, p(genes) = p-value controlling for position of genes calculated from 1,000 Monte-Carlo randomisations, p(immune genes) = p-value controlling for position of immune-related genes calculated from 1,000 Monte-Carlo randomisations. P(genes) and p(immune genes) are calculated only for enrichment with uncontrolled p<0.05. Click here for file

Additional file 9: Table S5 Enrichment of autoimmune disease associated regions with and without other transcription factors present at each VDR binding site. O/E, = observed/expected overlap of genomic intervals; P, = P-value calculated from 10,000 Monte-Carlo randomizations. Click here for file

Additional file 10: Figure S5 Overlap with autoimmune disease association regions with variable distances around autoimmune single nucleotide polymorphisms. Enrichment is shown for different distances in base-pairs around SNPs from genome-wide association studies (GWAS) implicated in autoimmune diseases with *P*<10^-7^[27]. Click here for file

Additional file 11: Table S4 Autoimmune disease-associated single nucleotide polymorphisms located within VDR ChIP-seq peaks. RegulomeDB score: 1a eQTL + TF binding + matched TF motif + matched DNase Footprint + DNase peak; 1b eQTL + TF binding + any motif + DNase Footprint + DNase peak; 1c eQTL + TF binding + matched TF motif + DNase peak; 1d eQTL + TF binding + any motif + DNase peak; 1e eQTL + TF binding + matched TF motif; 1f eQTL + TF binding/DNase peak; 2a TF binding + matched TF motif + matched DNase Footprint + DNase peak; 2b TF binding + any motif + DNase Footprint + DNase peak; 2c TF binding + matched TF motif + DNase peak; 3a TF binding + any motif + DNase peak; 3b TF binding + matched TF motif; 4 TF binding + DNase peak; 5 TF binding or DNase peak; 6 other; 7 no functional annotation.25(OH)D≥75 = samples with 25-hydroxyvitamin D ≥75 nM, 25(OH)D<75 = samples with 25-hydroxyvitamin D <75 nM, TF = transcription factor. Click here for file
