# Supplementary figures and images for: Vitamin D receptor ChIP-seq in primary CD4+ cells: relationship to serum 25-hydroxyvitamin D levels and autoimmune disease
Source: BMC Med. 2013 Jul 12;11:163. doi: 10.1186/1741-7015-11-163 (PMC3710212; doi:10.1186/1741-7015-11-163)

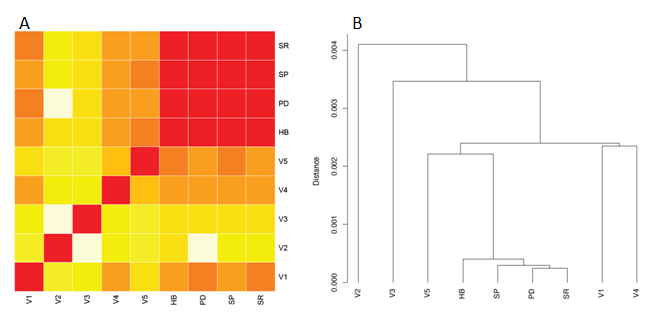

Supplement: Additional file 2: Figure S1 — Heirarchical clustering of VDR ChIP-seq peaks for individual samples. (A) Distance matrix computing distances as the inverse of overlap-enrichment pairwise similarity between samples (color scheme ranges from red for most similar to white for least similar). (B) Dendrogram incorporating the distances from the distance matrix. V1-5, VDR_1 to VDR_5; 25-hydroxyvitamin D ≥75 nM. HB, PD, SP and SR, 25-hydroxyvitamin D <75 nM. [file 1741-7015-11-163-S2.tif]

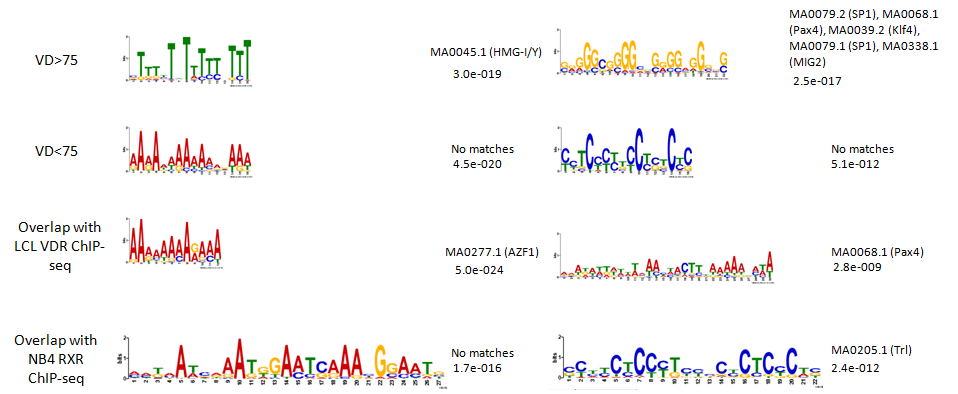

Supplement: Additional file 4: Figure S2 — Top MEME-ChIP motifs for VDR ChIP-seq peaks. This figure shows the top two motifs for each set of VDR ChIP-seq peaks by E-score as established by MEME-ChIP [15]: VD ≥ 75, samples with 25-hydroxyvitamin D ≥75 nM; VD <75, samples with 25-hydroxyvitamin D <75 nM. CD4+ VDR ChIP-seq peaks overlapping LCL VDR ChIP-seq peaks and CD4+ VDR ChIP-seq peaks overlapping NB4 RXR ChIP-seq peaks. [file 1741-7015-11-163-S4.tif]

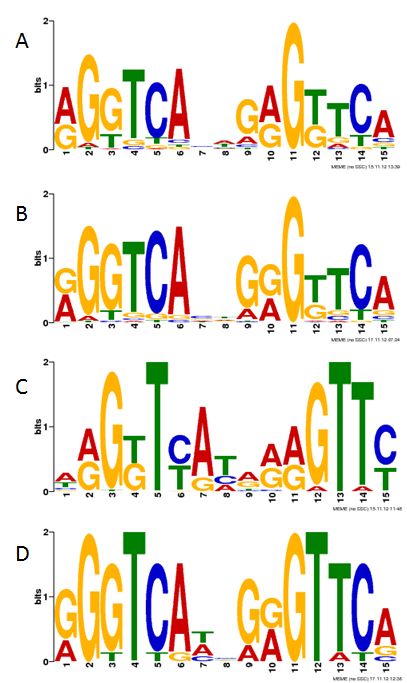

Supplement: Additional file 5: Figure S3 — Sequence logos for VDR-like binding site motifs. The motifs were identified for each data set by searching the full ChIP-Seq regions with the Jaspar/TRANSFAC RXRA::VDR motif using MAST, followed by de novo motif discovery with MEME on the positive regions from MAST [15]. The resulting VDR-like matrix was used for another round of MAST searching on the full ChIP-Seq regions and MEME motif discovery on the positive set. The final matrices are shown for (A) LCL (434 sites used by MEME), (B) MCL (288 sites), (C) CD4+ (56 sites), and (D) Jaspar/TRANSFAC RXRA::VDR. The observation that the LCL and MCL logos are more similar to each other than to the RXRA::VDR logo, whereas the logo for CD4+ is more similar to the RXRA::VDR logo, may reflect the fact that the two first logos are based on a much larger number of sites and are, therefore, more likely to represent the true binding site motif for strong binding. [file 1741-7015-11-163-S5.tif]

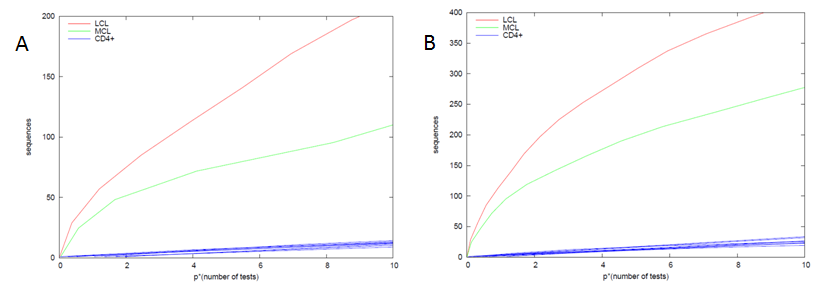

Supplement: Additional file 6: Figure S4 — Number of sequences retrieved from each data set by motif-based searches. Motif occurrences were identified using FIMO with (A) the RXRA::VDR motif, or (B) individually optimal matrices for each data set (LCL, MCL and CD4+) [20]. The number of sequences with at least one motif is plotted as a function of motif P-value. Each P-value is corrected for data set size by multiplying it with the number of tests. [file 1741-7015-11-163-S6.tif]

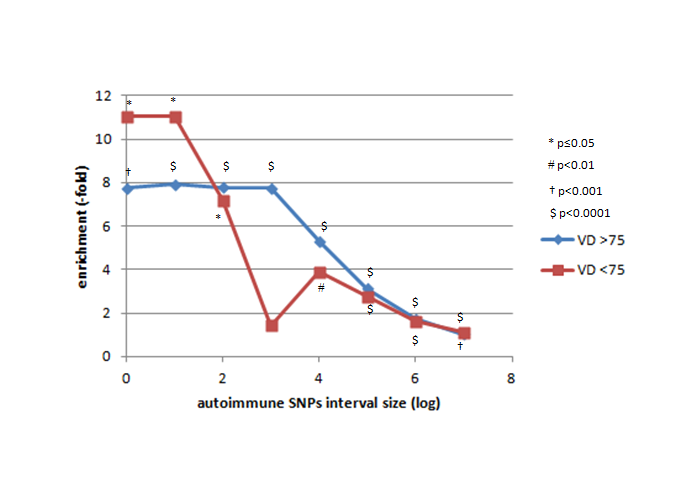

Supplement: Additional file 10: Figure S5 — Overlap with autoimmune disease association regions with variable distances around autoimmune single nucleotide polymorphisms. Enrichment is shown for different distances in base-pairs around SNPs from genome-wide association studies (GWAS) implicated in autoimmune diseases with P<10-7[27]. [file 1741-7015-11-163-S10.tif]
