# Supplementary material for: Vitamin D receptor ChIP-seq in primary CD4+ cells: relationship to serum 25-hydroxyvitamin D levels and autoimmune disease
Source: BMC Med. 2013 Jul 12;11:163. doi: 10.1186/1741-7015-11-163 (PMC3710212; doi:10.1186/1741-7015-11-163)
Supplement: Additional file 3: Table S1 — Top gene ontology terms by binomial FDR Q-value. Values are derived from GREAT. 25(OH)D≥75 = subjects with vitamin D levels ≥75 nM, 25(OH)D<75 = subjects with vitamin D levels <75 nM. [file 1741-7015-11-163-S3.doc]

**Table S1 Top gene ontology terms by binomial FDR Q-value.** Values are derived from GREAT (14). 25(OH)D≥75 = subjects with vitamin D levels ≥75nM, 25(OH)D<75 = subjects with vitamin D levels <75nM.

| Samples | GO Biological Pathway | Binomial p-value | Binomial FDR Q-value | Binomial enrichment |
| --- | --- | --- | --- | --- |
| 25(OH)D≥75 | mRNA metabolic process | 6.93E-159 | 3.04E-155 | 2.26 |
| 25(OH)D≥75 | RNA splicing, via transesterification reactions | 2.76E-150 | 8.06E-147 | 3.41 |
| 25(OH)D≥75 | nuclear mRNA splicing, via spliceosome | 4.93E-150 | 1.08E-146 | 3.43 |
| 25(OH)D≥75 | RNA processing | 2.54E-126 | 2.79E-123 | 2.08 |
| 25(OH)D≥75 | mRNA processing | 2.28E-121 | 2.21E-118 | 2.33 |
| 25(OH)D≥75 | RNA splicing | 2.61E-114 | 2.29E-111 | 2.49 |
| 25(OH)D≥75 | rRNA processing | 2.10E-93 | 9.21E-91 | 4.14 |
| 25(OH)D≥75 | rRNA metabolic process | 1.87E-87 | 7.79E-85 | 3.87 |
| 25(OH)D≥75 | ribosome biogenesis | 8.95E-82 | 3.56E-79 | 3.32 |
| 25(OH)D≥75 | protein folding | 1.43E-80 | 5.44E-78 | 2.63 |
| 25(OH)D≥75 | mRNA 3'-end processing | 1.39E-76 | 4.35E-74 | 4.45 |
| 25(OH)D≥75 | nuclear-transcribed mRNA poly(A) tail shortening | 4.11E-72 | 1.13E-69 | 7.22 |
| 25(OH)D≥75 | ncRNA processing | 3.23E-71 | 8.33E-69 | 2.69 |
| 25(OH)D≥75 | RNA 3'-end processing | 3.88E-70 | 9.45E-68 | 3.88 |
| 25(OH)D≥75 | nuclear-transcribed mRNA catabolic process, deadenylation-dependent decay | 1.54E-69 | 3.64E-67 | 4.56 |
| 25(OH)D≥75 | mRNA catabolic process | 2.11E-63 | 4.12E-61 | 3.25 |
| 25(OH)D≥75 | T cell activation | 1.99E-62 | 3.78E-60 | 2.26 |
| 25(OH)D≥75 | ribonucleoprotein complex biogenesis | 3.76E-62 | 6.86E-60 | 2.35 |
| 25(OH)D≥75 | cellular component biogenesis at cellular level | 2.41E-60 | 4.22E-58 | 2.29 |
| 25(OH)D≥75 | T cell differentiation | 3.82E-59 | 6.43E-57 | 2.53 |
| 25(OH)D<75 | nucleosome assembly | 1.59E-25 | 1.39E-21 | 5.34 |
| 25(OH)D<75 | chromatin assembly | 1.27E-23 | 5.55E-20 | 4.90 |
| 25(OH)D<75 | nucleosome organization | 1.52E-21 | 3.33E-18 | 4.38 |
| 25(OH)D<75 | chromatin assembly or disassembly | 5.10E-21 | 8.93E-18 | 4.27 |
| 25(OH)D<75 | protein-DNA complex assembly | 1.56E-20 | 2.28E-17 | 4.24 |
| 25(OH)D<75 | DNA packaging | 3.80E-20 | 4.75E-17 | 3.84 |
| 25(OH)D<75 | protein-DNA complex subunit organization | 9.84E-19 | 9.58E-16 | 3.83 |
| 25(OH)D<75 | DNA conformation change | 3.97E-17 | 3.48E-14 | 3.30 |
| 25(OH)D<75 | cellular macromolecular complex subunit organization | 8.89E-15 | 5.99E-12 | 2.07 |
| 25(OH)D<75 | viral reproduction | 1.76E-14 | 1.10E-11 | 2.45 |
| 25(OH)D<75 | translation | 3.88E-13 | 1.79E-10 | 2.45 |
| 25(OH)D<75 | cellular macromolecular complex assembly | 1.39E-12 | 5.28E-10 | 2.09 |
| 25(OH)D<75 | CenH3-containing nucleosome assembly at centromere | 1.03E-10 | 3.59E-08 | 7.51 |
| 25(OH)D<75 | chromatin remodeling at centromere | 5.67E-10 | 1.84E-07 | 6.73 |
| 25(OH)D<75 | ncRNA metabolic process | 5.74E-10 | 1.80E-07 | 2.36 |
| 25(OH)D<75 | protein peptidyl-prolyl isomerization | 1.33E-07 | 2.91E-05 | 4.05 |
| 25(OH)D<75 | nuclear mRNA splicing, via spliceosome | 1.36E-07 | 2.90E-05 | 2.29 |
| 25(OH)D<75 | histone exchange | 1.78E-07 | 3.63E-05 | 4.59 |
| 25(OH)D<75 | nuclear export | 2.01E-07 | 3.83E-05 | 3.24 |
| 25(OH)D<75 | RNA splicing, via transesterification reactions | 2.12E-07 | 3.95E-05 | 2.25 |
