# Supplementary material for: Vitamin D receptor ChIP-seq in primary CD4+ cells: relationship to serum 25-hydroxyvitamin D levels and autoimmune disease
Source: BMC Med. 2013 Jul 12;11:163. doi: 10.1186/1741-7015-11-163 (PMC3710212; doi:10.1186/1741-7015-11-163)
Supplement: Additional file 7: Table S2 — Enrichment of genomic features within VDR binding sites. This shows the enrichment within VDR binding intervals for VDR binding intervals in lymphoblastoid cell lines (LCL VDR) and monocytic cell lines (MCL VDR), and other genomic features drawn from Cistrome and ENCODE [18,25,44]. 25(OH)D≥75 = subjects with vitamin D levels ≥75 nM, 25(OH)D<75 = subjects with vitamin D levels <75 nM, O/E = observed/expected overlap of genomic intervals, p = p-value calculated from 10,000 Monte-Carlo randomisations. [file 1741-7015-11-163-S7.doc]

**Table S2 Enrichment of genomic features within VDR binding sites.** This shows the enrichment within VDR binding intervals for VDR binding intervals in lymphoblastoid cell lines (LCL VDR) and monocytic cell lines (MCL VDR), and other genomic features drawn from Cistrome and ENCODE (18, 25, 44). 25(OH)D≥75 = subjects with vitamin D levels ≥75nM, 25(OH)D<75 = subjects with vitamin D levels <75nM, O/E = observed/expected overlap of genomic intervals, p = p-value calculated from 10,000 Monte-Carlo randomisations.

| Genomic feature | 25(OH)D≥75 | | 25(OH)D<75 | | 25(OH)D≥75 vs. 25(OH)D<75 |
| --- | --- | --- | --- | --- | --- |
|  | O/E | p | O/E | p | p |
| LCL VDR | 70.0 | 0.0001 | 151.7 | 0.0001 | 0.0002 |
| MCL VDR | 28.8 | 0.0001 | 37.2 | 0.0001 | 0.0002 |
| DNase I peaks | 18.9 | 0.0001 | 23.7 | 0.0001 | 0.0002 |
| ETS1 ChIP-seq (GM12878) | 145.4 | 0.0001 | 373.5 | 0.0001 | 0.0164 |
| Pol2 ChIP-seq (GM12878) | 37.3 | 0.0001 | 50.2 | 0.0001 | 0.0002 |
| SP1 ChIP-seq (GM12878) | 45.9 | 0.0001 | 76.8 | 0.0001 | 0.0002 |
| CTCF ChIP-seq (K562) | 22.3 | 0.0001 | 17.2 | 0.0001 | 0.0002 |
| NR4A1 ChIP-seq (K562) | 12.5 | 0.0001 | 19.4 | 0.0001 | 0.0004 |
| c-MYC (K562) | 83.9 | 0.0001 | 155.4 | 0.0001 | 0.0002 |
| RXR ChIP-seq (NB4) | 19.8 | 0.0004 | 65.1 | 0.0001 | 0.0238 |
| H3K27Ac (GM12878) | 11.3 | 0.0001 | 12.8 | 0.0001 | 0.0002 |
| H2A.Z (GM12878) | 6.6 | 0.0001 | 7.4 | 0.0001 | 0.0002 |
| H3K4me1 (GM12878) | 4.9 | 0.0001 | 4.1 | 0.0001 | 0.0002 |
| H3K4me2 (GM12878) | 9.8 | 0.0001 | 10.2 | 0.0001 | 0.0002 |
| H3K4me3 (GM12878) | 10.8 | 0.0001 | 11.8 | 0.0001 | 0.0002 |
| H3K9Ac (GM12878) | 14.0 | 0.0001 | 15.7 | 0.0001 | 0.0002 |
| H3K9me3 (GM12878) | 1.3 | 0.0001 | 1.3 | 0.0001 | 0.0002 |
