# Supplementary material for: Vitamin D receptor ChIP-seq in primary CD4+ cells: relationship to serum 25-hydroxyvitamin D levels and autoimmune disease
Source: BMC Med. 2013 Jul 12;11:163. doi: 10.1186/1741-7015-11-163 (PMC3710212; doi:10.1186/1741-7015-11-163)
Supplement: Additional file 8: Table S3 — Enrichment of autoimmune disease susceptibility regions within VDR binding sites. Autoimmune susceptibility regions were defined as those within 100kb of SNPs associated with autoimmune disease [27]. 25(OH)D≥75 = subjects with vitamin D levels ≥75 nM, 25(OH)D<75 = subjects with vitamin D levels <75 nM, O/E = observed/expected overlap of genomic intervals, p = p-value calculated from 10,000 Monte-Carlo randomisations, p(genes) = p-value controlling for position of genes calculated from 1,000 Monte-Carlo randomisations, p(immune genes) = p-value controlling for position of immune-related genes calculated from 1,000 Monte-Carlo randomisations. P(genes) and p(immune genes) are calculated only for enrichment with uncontrolled p<0.05. [file 1741-7015-11-163-S8.doc]

**Table S3 Enrichment of autoimmune disease susceptibility regions within VDR binding sites.** Autoimmune susceptibility regions were defined as those within 100kb of SNPs associated with autoimmune disease (27). 25(OH)D≥75 = subjects with vitamin D levels ≥75nM, 25(OH)D<75 = subjects with vitamin D levels <75nM, O/E = observed/expected overlap of genomic intervals, p = p-value calculated from 10,000 Monte-Carlo randomisations, p(genes) = p-value controlling for position of genes calculated from 1,000 Monte-Carlo randomisations, p(immune genes) = p-value controlling for position of immune-related genes calculated from 1,000 Monte-Carlo randomisations. P(genes) and p(immune genes) are calculated only for enrichment with uncontrolled p<0.05.

| Disease | 25(OH)D≥75 | | | | 25(OH)D<75 | | | | 25(OH)D≥75 vs. 25(OH)D<75 |
| --- | --- | --- | --- | --- | --- | --- | --- | --- | --- |
|  | O/E | p | p(genes) | p(immune genes) | O/E | p | p(genes) | p(immune genes) | p |
| Alopecia | 4.07 | 0.0176 | 0.002 | 0.4695 | 3.63 | 0.1096 | N/A | N/A | 0.3168 |
| Ankylosing spondylitis | 3.19 | 0.004 | 0.001 | 0.002 | 0.28 | 0.6613 | N/A | N/A | 0.0852 |
| JIA | 2.00 | 0.192 | N/A | N/A | 6.51 | 0.0623 | N/A | N/A | 1 |
| Behcet's disease | 0.59 | 0.4348 | N/A | N/A | 0.00 | 1.0000 | N/A | N/A | 1 |
| Celiac disease | 3.17 | 0.0002 | 0.001 | 0.002 | 1.39 | 0.2872 | N/A | N/A | 0.002 |
| Crohn's disease | 2.79 | 0.0001 | 0.001 | 0.001 | 2.37 | 0.0174 | 0.01 | 0.223 | 0.0016 |
| Grave's disease | 7.97 | 0.0001 | 0.001 | 0.001 | 5.24 | 0.0365 | 0.035 | 0.186 | 0.5263 |
| Kawasaki disease | 2.52 | 0.2163 | N/A | N/A | 6.35 | 0.0876 | N/A | N/A | 0.8421 |
| Multiple sclerosis | 4.60 | 0.0001 | 0.001 | 0.001 | 3.29 | 0.0052 | 0.005 | 0.049 | 0.001 |
| Primary biliary cirrhosis | 4.34 | 0.0001 | 0.001 | 0.001 | 2.95 | 0.0524 | N/A | N/A | 0.3002 |
| Primary sclerosing cholangitis | 0.55 | 0.3923 | N/A | N/A | 0.00 | 1.0000 | N/A | N/A | 1 |
| Psoriasis | 3.54 | 0.0001 | 0.001 | 0.002 | 4.04 | 0.009699 | 0.023 | 0.163 | 0.4072 |
| Psoriatic arthritis | 5.85 | 0.0008 | 0.001 | 0.001 | 2.09 | 0.1472 | N/A | N/A | 0.5897 |
| Rheumatoid arthritis | 2.56 | 0.0009 | 0.001 | 0.284 | 2.62 | 0.0381 | 0.051 | 0.595 | 0.0134 |
| Systemic lupus erythematosus | 4.33 | 0.0001 | 0.001 | 0.001 | 2.87 | 0.0271 | 0.023 | 0.260 | 0.0032 |
| Systemic sclerosis | 3.23 | 0.011 | 0.001 | 0.1788 | 0.66 | 0.5097 | N/A | N/A | 0.05219 |
| Type 1 diabetes mellitus | 3.75 | 0.0001 | 0.001 | 0.001 | 3.34 | 0.0109 | 0.005 | 0.08 | 0.0344 |
| Ulcerative colitis | 1.59 | 0.0038 | 0.001 | 0.01614 | 1.93 | 0.05489 | N/A | N/A | 0.3642 |
| Vitiligo | 4.22 | 0.0001 | 0.001 |  | 5.51 | 0.009899 | 0.001 | 0.005 | 0.6191 |
|  |  |  |  |  |  |  |  |  |  |
| Autoimmune disease (pooled) | 3.13 | 0.0001 | 0.001 | 0.001 | 2.76 | 0.0001 | 0.001 | 0.005 | 0.0002 |
