# Supplementary material for: Vitamin D receptor ChIP-seq in primary CD4+ cells: relationship to serum 25-hydroxyvitamin D levels and autoimmune disease
Source: BMC Med. 2013 Jul 12;11:163. doi: 10.1186/1741-7015-11-163 (PMC3710212; doi:10.1186/1741-7015-11-163)
Supplement: Additional file 9: Table S5 — Enrichment of autoimmune disease associated regions with and without other transcription factors present at each VDR binding site. O/E, = observed/expected overlap of genomic intervals; P, = P-value calculated from 10,000 Monte-Carlo randomizations. [file 1741-7015-11-163-S9.doc]

**Table S5. Enrichment of autoimmune disease associated regions with and without other transcription factors present at each VDR binding site. O/E, = observed/expected overlap of genomic intervals;, P, = P-value calculated from 10,000 Monte-Carlo randomizations.**

| VDR binding sites overlapping with other transcription factor (TFs) binding sites | VDR peaks overlapping with TFs | | VDR peaks not overlapping with TFs | | Overlapping vs. non-overlapping VDR peaks |
| --- | --- | --- | --- | --- | --- |
|  | O/E | P | O/E | P | P |
| ETS1, SP1, CTCF, NR4A1, c-MYC, RXR | 3.03 | 0.0001 | 3.15 | 0.0001 | 0.27 |
| ETS1 (GM12878) | 1.74 | 0.45 | 3.10 | 0.0001 | 0.26 |
| SP1 (GM12878) | 3.01 | 0.0001 | 3.12 | 0.0001 | 0.09 |
| NR4A1 (K562) | 2.70 | 0.01 | 3.10 | 0.0001 | 0.71 |
| CTCF (K562) | 3.26 | 0.0001 | 3.03 | 0.0001 | 0.25 |
| c-MYC (K562) | 2.54 | 0.0001 | 3.17 | 0.0001 | 0.42 |
| RXR (NB4) | 1.74 | 0.15 | 3.13 | 0.0001 | 0.41 |
