# Supplementary material for: Vitamin D receptor ChIP-seq in primary CD4+ cells: relationship to serum 25-hydroxyvitamin D levels and autoimmune disease
Source: BMC Med. 2013 Jul 12;11:163. doi: 10.1186/1741-7015-11-163 (PMC3710212; doi:10.1186/1741-7015-11-163)
Supplement: Additional file 11: Table S4 — Autoimmune disease-associated single nucleotide polymorphisms located within VDR ChIP-seq peaks. RegulomeDB score: 1a eQTL + TF binding + matched TF motif + matched DNase Footprint + DNase peak; 1b eQTL + TF binding + any motif + DNase Footprint + DNase peak; 1c eQTL + TF binding + matched TF motif + DNase peak; 1d eQTL + TF binding + any motif + DNase peak; 1e eQTL + TF binding + matched TF motif; 1f eQTL + TF binding/DNase peak; 2a TF binding + matched TF motif + matched DNase Footprint + DNase peak; 2b TF binding + any motif + DNase Footprint + DNase peak; 2c TF binding + matched TF motif + DNase peak; 3a TF binding + any motif + DNase peak; 3b TF binding + matched TF motif; 4 TF binding + DNase peak; 5 TF binding or DNase peak; 6 other; 7 no functional annotation.25(OH)D≥75 = samples with 25-hydroxyvitamin D ≥75 nM, 25(OH)D<75 = samples with 25-hydroxyvitamin D <75 nM, TF = transcription factor. [file 1741-7015-11-163-S11.doc]

**Table S4 Autoimmune disease-associated single nucleotide polymorphisms located within VDR ChIP-seq peaks. RegulomeDB score: 1a eQTL + TF binding + matched TF motif + matched DNase Footprint + DNase peak; 1b eQTL + TF binding + any motif + DNase Footprint + DNase peak; 1c eQTL + TF binding + matched TF motif + DNase peak; 1d eQTL + TF binding + any motif + DNase peak; 1e eQTL + TF binding + matched TF motif; 1f eQTL + TF binding / DNase peak; 2a TF binding + matched TF motif + matched DNase Footprint + DNase peak; 2b TF binding + any motif + DNase Footprint + DNase peak; 2c TF binding + matched TF motif + DNase peak; 3a TF binding + any motif + DNase peak; 3b TF binding + matched TF motif; 4 TF binding + DNase peak; 5 TF binding or DNase peak; 6 other; 7 no functional annotation (28). 25(OH)D≥75 = samples with 25-hydroxyvitamin D ≥75nM, 25(OH)D<75 = samples with 25-hydroxyvitamin D <75nM, TF = transcription factor.**

| dbSNP ID | Chromosome | Position | Group | Associated diseases | TF motif | ENCODE TF ChIP-seq peak | eQTL | Regulome DB score |
| --- | --- | --- | --- | --- | --- | --- | --- | --- |
| rs7329174 | chr13 | 41558110 | 25(OH)D≥75 | Systemic lupus erythematosus | None | RFX3 | None | 4 |
| rs4763879 | chr12 | 9910164 | 25(OH)D≥75 | Type 1 diabetes mellitus | P50:P50 | POLR2A, CREBBP | CLEC2B, CLEC2D, DCAL1 | 1f |
| rs907611 | chr11 | 1874072 | 25(OH)D≥75 | Ulcerative colitis | YY1, NF-muE1 | YY1, GABPA | None | 2a |
| rs1465788 | chr14 | 69263599 | 25(OH)D≥75 | Type 1 diabetes mellitus | None | CTCF, CREBBP | None | 4 |
| rs9468925 | chr6 | 31258837 | 25(OH)D≥75 | Vitiligo | MECP2 | None | HLA-C | 6 |
| rs5029939 | chr6 | 138195723 | 25(OH)D≥75 | Systemic lupus erythematosus | None | POLR2A | None | 5 |
| rs610604 | chr6 | 138199417 | 25(OH)D≥75 | Psoriasis | None | SPI1 | None | 4 |
| rs1738074 | chr6 | 159465977 | 25(OH)D≥75 | Multiple sclerosis, celiac disease | None | EBF1, POLR2A, POU2F2, RAD21, TBP, TAF1, NFKB1, MAX | None | 4 |
| rs6451493 | chr5 | 40410935 | 25(OH)D≥75 | Ulcerative colitis | Tcfap2e | SMARCA4, MEIS1, FOS, JUN, JUND, USF2 | None | 3a |
| rs11742570 | chr5 | 40410584 | 25(OH)D≥75 | Crohn's disease | None | SMARCA4, GATA2, MEIS1 | None | 4 |
| rs6832151 | chr4 | 40303633 | 25(OH)D≥75 | Grave's disease | None | POLR2A, ELF1, TBP | None | 4 |
| rs7665090 | chr4 | 103551603 | 25(OH)D≥75 | Primary biliary cirrhosis | Pit-1 | None | MANBA | 6 |
| rs35675666 | chr1 | 8021973 | 25(OH)D≥75 | Ulcerative colitis | None | POLR2A, YY1, ETS1, EGR1, SIN3A, ELF1, TAF1, E2F1, CCNT2, MYC, MAX, CDX2, CREBBP, GATA1 | None | 4 |
| rs6451493 | chr5 | 40410935 | 25(OH)D<75 | Ulcerative colitis | Tcfap2e | SMARCA4, MEIS1, FOS, JUN, JUND, USF2 | None | 3a |
| rs11742570 | chr5 | 40410584 | 25(OH)D<75 | Crohn's disease | None | SMARCA4, GATA2, MEIS1 | None | 4 |
